# Quantification of hCG Hormone Using Tapered Optical Fiber Decorated with Gold Nanoparticles

**DOI:** 10.3390/s23208538

**Published:** 2023-10-18

**Authors:** David Saúl Villegas-Cantoran, Celia Lizeth Gómez, Luz del Carmen Gómez-Pavón, Placido Zaca-Morán, Dulce Natalia Castillo-López, Arnulfo Luis-Ramos, Jesús Manuel Muñoz-Pacheco

**Affiliations:** 1 Grupo de Sistemas Fotónicos y Nanoóptica, Facultad de Ciencias de la Electrónica, Benemérita Universidad Autónoma de Puebla, Puebla 72570, Mexico; david.villegasc@alumno.buap.mx (D.S.V.-C.); dulce.castillolo@correo.buap.mx (D.N.C.-L.); arnulfo.luis@correo.buap.mx (A.L.-R.); jesusm.pacheco@correo.buap.mx (J.M.M.-P.); 2 Instituto de Ciencias, Ecocampus Valsequillo, Benemérita Universidad Autónoma de Puebla, Puebla 72570, Mexico; celia.gomez@correo.buap.mx (C.L.G.); placido.zaca@correo.buap.mx (P.Z.-M.)

**Keywords:** evanescent field, tapered optical fiber, hCG hormone, gold nanoparticles

## Abstract

In this study, a novel technique for the quantification of the human chorionic gonadotropin (hCG) hormone using localized surface plasmons and a tapered optical fiber decorated with gold nanoparticles (Au-NPs) is reported. The tapered optical fiber fabrication process involves stretching a single-mode optical fiber using the flame-brushing system. The waist of the tapered optical fiber reaches a diameter of 3 μm. Decoration of the taper is achieved through the photodeposition of 100 nm Au-NPs using the drop-casting technique and a radiation source emitting at 1550 nm. The presence of the hCG hormone in the sample solutions is verified by Fourier-transform infrared spectroscopy (FTIR), revealing the presence of bands related to functional groups, such as C=O (1630 cm${}^{-1}$), which are associated with proteins and lipids, components of the hCG hormone. Quantification tests for hormone concentrations were carried out by measuring the optical power response of the tapered optical fiber with Au-NPs under the influence of hCG hormone concentrations, ranging from 1 mIU/mL to 100,000 mIU/mL. In the waist of the tapered optical fiber, the evanescent field is amplified because of localized surface plasmons generated by the nanoparticles and the laser radiation through the optical fiber. Experimental results demonstrated a proportional relationship between measured radiation power and hCG concentration, with the optical power response decreasing from 4.45 mW down to 2.5 mW, as the hCG hormone concentration increased from 1 mIU/mL up to 100,000 mIU/mL. Furthermore, the spectral analysis demonstrated a spectral shift of 14.2 nm with the increment of the hCG hormone concentration. The measurement system exhibits promising potential as a sensor for applications in the biomedical and industrial fields.

## 1. Introduction

The measurement of different concentrations of the hCG hormone plays a significant role in detecting various stages of pregnancy and serves as a potential biomarker for the detection and localization of different types of cancers, such as breast [1,2,3], prostate [4,5,6], and pancreatic cancer [7,8,9,10], which have high mortality rates due to their detection at advanced stages of the disease. Typically, hCG hormone detection is carried out using electrochemical sensors [11,12,13,14]. However, these types of sensors require a large volume of biological samples for analysis, are susceptible to high electromagnetic interference that can result in false positives, and are expensive to manufacture. Additionally, there are piezoelectric sensors that offer high sensitivity and a compact size, but have drawbacks, such as a very low output response, requiring amplification stages for proper interpretation [15].

Quantification and detection systems, especially those based on optical fiber, are gaining significant importance in hCG hormone detection [16,17,18]. These sensors offer several advantages compared to other types, such as high sensitivity [19], real-time monitoring capability [20], immunity to electromagnetic interference [21,22], low manufacturing cost [23], detection of very low concentrations [24], and compact sizes, making them adaptable to various hazardous installations [25].

In 2018, a method for detecting the hCG hormone using electrochemical transducers, aided by lateral flow analysis (LFA) test strips and a transducer, was reported. The system mainly consists of two copper electrodes attached to the LFA strips, allowing them to measure concentrations of 25 mIU/mL, 100 mIU/mL, 200 mIU/mL, 1000 mIU/mL, 5000 mIU/mL, and 10,000 mIU/mL. Measurements of resistivity changes upon interaction with the bioreceptor (hCG antigen) showed changes of 25 mIU/mL for every 10 MΩ ± 0.01, indicating that this system could detect a low concentration of the hCG hormone [26].

In 2020, a study on hCG hormone detection using a single-mode D-shaped hollow-core optical fiber as a transducer was conducted [11]. To create the sensor, a D-shaped optical fiber system was developed by fusing two single-mode optical fibers with a single-mode hollow-core optical fiber at its core, followed by precision polishing of the hollow-core fiber’s diameter to 26.5 μm. Subsequently, this sensor was immersed in solutions containing three distinct hCG hormone concentrations (5 mIU/mL, 50 mIU/mL, and 100 mIU/mL) and its response was studied through a spectrometer. The experimental findings, based on four repetitions for each concentration, revealed a direct correlation between the hCG hormone concentration and frequency shift, with an observed shift of approximately 0.82 nm, extending up to 100 mIU/mL.

The use of fiber optic technology and the study of the surface plasmon resonance (SPR) phenomenon have improved the detection of optical parameters in biological solutions due to their high real-time sensitivity, particularly in medical applications [27,28,29]. In a recent study by Esmailidastjerdipour et al. in 2023 [30], the diagnosis of tuberculosis through changes in the refractive index of blood plasma was reported using an SPR-based fiber optic sensor. The numerical results revealed that selecting a fiber optic sensor structure incorporating different aluminum/copper alloy compositions in a 30/70 ratio can significantly enhance both sensitivity and detection accuracy. It was determined that when the real part of the dielectric coefficient is higher, the surface plasmon wave vector strengthens, making the structure more sensitive to small changes in the refractive index of the detection medium (blood plasma). In this context, it was concluded that the structure combining aluminum and copper alloy at this specific ratio is the most effective option for improving the sensor’s quality in the early detection of tuberculosis.

The main drawbacks of the systems reported so far include the use of multiple chemical processes for optical fiber functionalization. In the case of electrochemical transducers, their manufacturing involves several costly stages, complex electronic instrumentation is required to monitor the output response, and specialized laboratories with controlled environments are necessary. In this study, we report on a new method for quantifying the hCG hormone using localized surface plasmons with a specially prepared tapered optical fiber coated with gold nanoparticles. The fabrication process involves stretching a single-mode optical fiber to a diameter of 3 µm using a flame-brushing system, followed by decorating the taper with 100 nm Au-NPs using the drop-casting technique and a 1550 nm laser source. The developed measurement system shows promising potential for applications in both biomedical and industrial settings.

## 2. Experimental Setup

### 2.1. Tapered Optical Fiber Fabrication

**Figure 1:**
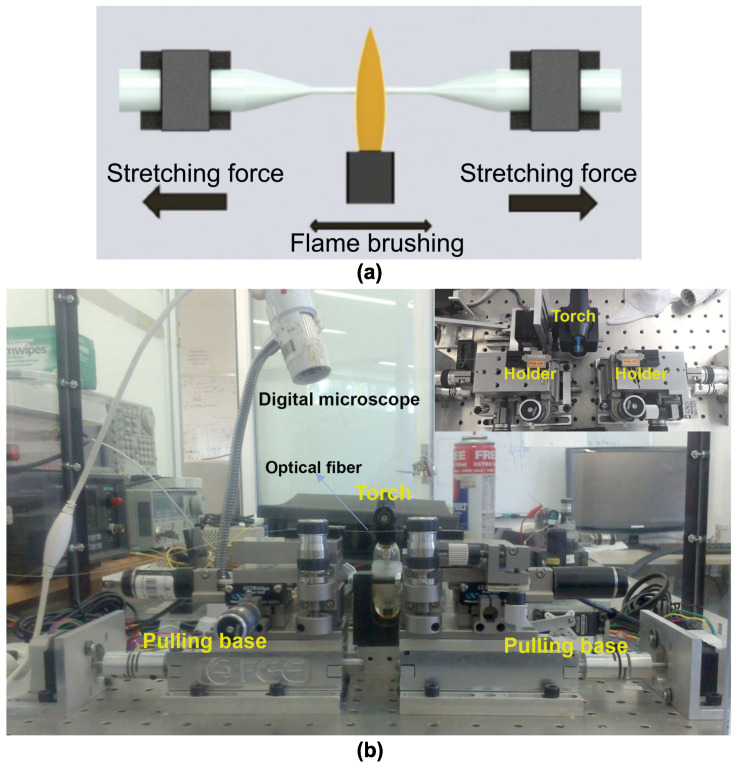
(**a**) Diagram of the flame-brushing technique. (**b**) Experimental setup for the fabrication of the tapered optical fiber.

The tapered optical fiber was fabricated using a single-mode optical fiber, SMF-28, using the flame-brushing technique shown in Figure 1a. Basically, this technique was carried out by an automatized mechatronic system that used a butane gas burner to create the thin zone on the optical fiber. The main components of the mechatronic system are a heating stage, stretching stage, and electronic control systems. In the stretching process, the coating of a standard optical fiber was initially removed; then, the bare optical fiber was cleaned with isopropyl alcohol and dried with a nitrogen gun. The optical fiber was then secured in the fiber holders and mounted to the pulling bases, which control the stretching. Finally, a computerized control system was used to specify important fabrication parameters, such as the stretching length, speed of the pulling bases, and speed of oscillation for the burner. Using the aforementioned fabrication process, tapered optical fibers with diameters of less than 5 μm were obtained [31]. In Figure 1b, a photograph of the experimental setup used for the fabrication of taper fibers is shown.

### 2.2. Tapered Optical Fiber Decoration

**Figure 2:**
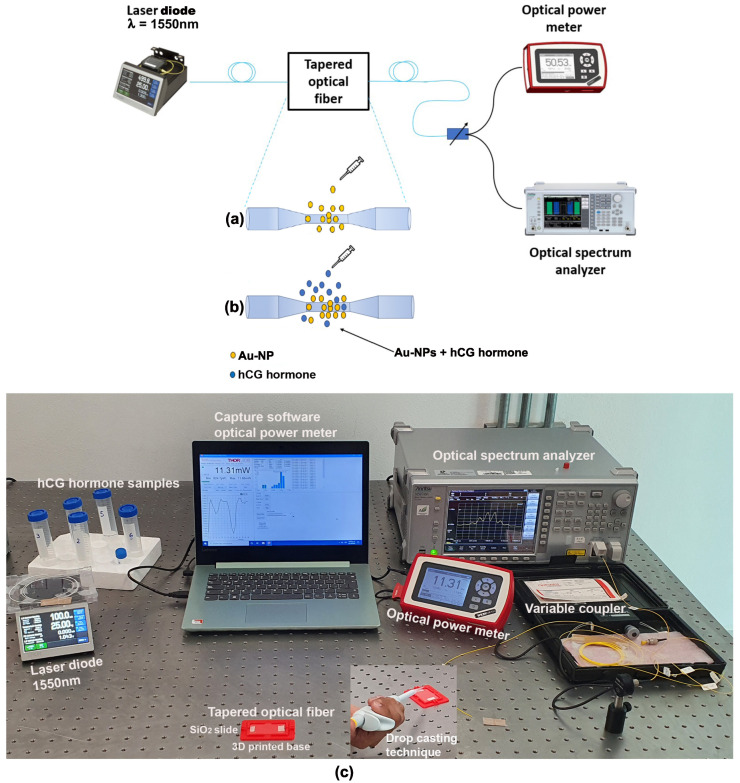
Experimental setup scheme for (**a**) Au-NPs and (**b**) hCG hormone photodeposition. (**c**) Experimental setup real-time photo (inset: drop-casting technique).

The optical setup arrangement for the tapered optical fiber decoration involving drop-casting and laser radiation techniques is shown in Figure 2. The radiation source used was a laser diode at 1550 nm (Model FPL1009S, Thorlabs, Newton, NJ, USA). The technique of photodeposition of metallic nanoparticles onto the optical fiber involves applying a solution containing Au-NPs to the fiber surface and using a coherent radiation source to achieve precise and controlled deposition. Notably, a 1550 nm radiation source was chosen due to the optical fiber’s ability to minimize absorption losses during propagation, resulting in higher energy transmission efficiency.

An optical power meter (Model PM100D, Thorlabs) was connected to the end of the tapered optical fiber to monitor the optical power propagating through it, alongside a spectrum analyzer (Model MS9740A, Anritsu, Kanagawa, JP), to obtain the spectral response of the samples. Optical radiation of approximately 34 mW was supplied into the tapered optical fiber, which was positioned on a glass slide. Subsequently, the taper waist was decorated with 100 nm Au-NPs through the withdrawal of 0.2 mL of Au-NPs suspended in citrate buffer (742031, Sigma-Aldrich, St. Louis, MO, USA). This solution was then introduced into an ultrasonic bath for homogenization. A droplet of the Au-NPs solution (0.7 μL) was carefully placed on the waist region of the tapered optical fiber using a micropipette (Figure 2a) [32]. Once the droplet with the Au-NPs solution had dried on the optical fiber, the output optical power measured was 5.5 mW.

**Figure 3:**
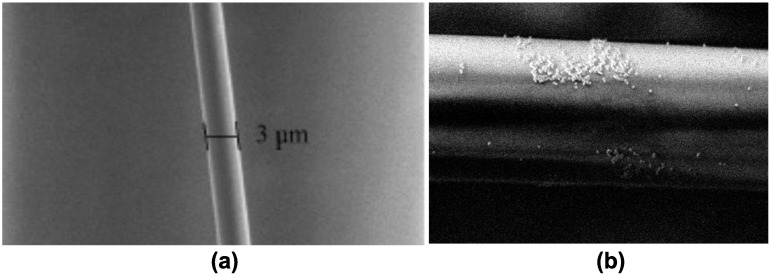
SEM micrograph of (**a**) the tapered optical fiber with a diameter of 3 µm and (**b**) the tapered optical fiber decorated with Au-NPs.

Figure 3 shows micrographs of the tapered optical fibers, characterized using scanning electron microscopy (SEM) under low vacuum conditions with a backscattered electron detector. Figure 3a shows the taper obtained through the flame-brushing technique, where it can be observed that the central part of the taper exhibits a uniform section, free from particles and defects in its longitudinal profile. On the other hand, in Figure 3b, the taper obtained after the decoration process with Au-NPs using the photodeposition technique is displayed. The image depicts a cluster of gold particles agglomerated on the fiber surface, distributed similarly throughout the cross-sectional area of the fiber.

### 2.3. Characterization of the hCG Hormone

A solution of 0.1 mg of the hCG hormone (lyophilized powder, a vial of ∼2500 IU, Sigma Aldrich) was prepared in 10 mL of distilled water and placed in an ultrasonic bath for 30 s to homogenize the solution. From this solution, six samples with the following hCG hormone concentrations were prepared: 1 mIU/mL, 10 mIU/mL, 100 mIU/mL, 1000 mIU/mL, 10,000 mIU/mL, and 100,000 mIU/mL.

As mentioned before, the optical power through the tapered optical fiber with Au-NPs was 34 mW, with an output optical power of 5.5 mW. Then, a drop of each hCG hormone sample (approximately 50 μL) was deposited on the waist of the tapered optical fiber decorated with Au-NPs (Figure 3b). The output optical power response was measured in each case. It is important to mention that during the experiments, a 50 μL drop of pure distilled water was tested on the tapered optical fiber decorated with Au-NPs. The optical power response observed was less than 1.5 mW, which is outside the range seen in the case of the prepared solutions with the hCG hormone.

**Figure 4:**
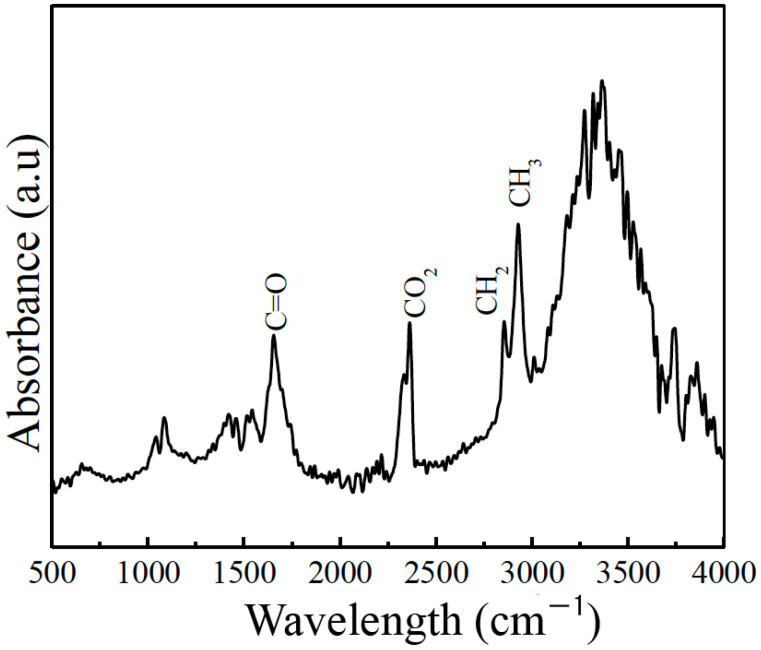
Characteristic FTIR spectrum of hCG hormone solutions.

To verify the presence of the hCG hormone in the prepared samples, Fourier-transform infrared spectroscopy and fluorescence spectroscopy were applied using a Fourier-transform infrared spectroscopy (FTIR) in the range of 4000 to 500 cm${}^{-1}$ using an attenuated total reflection (ATR) mode spectrometer (Bruker Vertex 70, Ettlingen, DEU). For this analysis, 1 μL of the hCG samples was placed and evaporated on the ATR crystal at room temperature for 15 min, which was used as the baseline. Figure 4 displays the characteristic spectrum of the samples, revealing the presence of bands related to functional groups, such as C=O (1630 cm${}^{-1}$), associated with proteins and lipids, characteristic of components of the hCG hormone. Additionally, a band of around 2348 cm${}^{-1}$ corresponding to the O=C=O bond was identified, along with bands linked to CH${}_{2}$ and CH${}_{3}$ groups at approximately 2850 and 2910 cm${}^{-1}$, respectively [33], indicating the presence of lipids. Furthermore, an intense band at around 3362 cm${}^{-1}$, corresponding to a combination of OH and NH bonds related to amino acids, was detected. All the identified bonds correspond to the main components of the hCG hormone.

**Figure 5:**
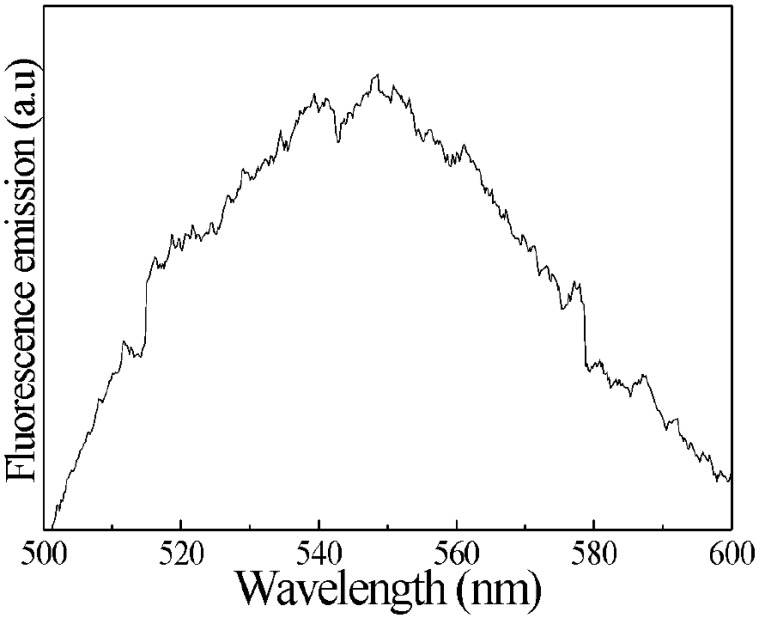
Characteristic fluorescence spectrum of hCG hormone solutions.

Furthermore, the analysis through fluorescence spectroscopy was performed within a wavelength range of 350 to 1050 nm using a spectrophotometer (Ocean Optics) equipped with a 365 nm excitation lamp. For this analysis, 500 μL of the hCG solution was employed. In the fluorescence spectrum (Figure 5), a distinctive band of around 543 nm was observed, indicating the presence of the hCG hormone in the analyzed solutions [34].

## 3. Results

In the waist of the tapered optical fiber, the electric field of the evanescent wave amplifies due to the localized surface plasmons (LSPs) generated by the nanoparticles and laser radiation [35].

**Figure 6:**
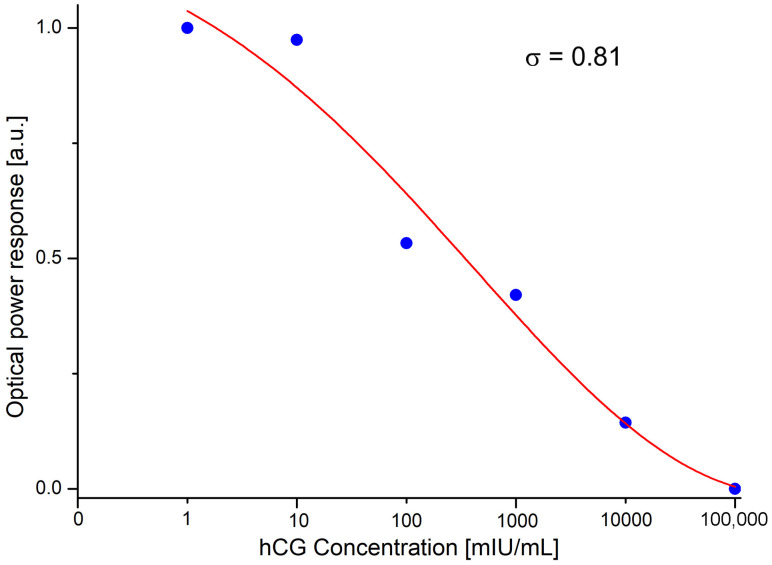
Optical power response measured as a function of the hCG concentration.

Figure 6 shows the normalized results obtained measuring different prepared concentrations of the hCG hormone at 1 mIU/mL, 10 mIU/mL, 100 mIU/mL, 1000 mIU/mL, 10,000 mIU/mL, and 100,000 mIU/mL. The optical power response decreases as the hCG hormone concentration increases.

In the experiments, it was observed that as soon as the amplified evanescent wave interacts with the hCG hormone, an immediate optical power response is generated. This response is observed from the optical output power of 5.5 mW mentioned above, obtaining the following: 4.45 mW for 1 mIU/mL, 4.4 mW for 10 mIU/mL, 3.54 mW for 100 mIU/mL, 3.32 mW for 1000 mIU/mL, 2.78 mW for 10,000 mIU/mL, and 2.5 mW for 100,000 mIU/mL. The calculated standard deviation is σ=0.81, with a coefficient of variation of 23%. This value falls within the range indicative of low variation.

The results obtained show that at lower concentrations of the hCG hormone, the normalized optical power response is higher and gradually decreases as the hCG concentration increases. This suggests an inverse relationship between the hCG concentration and the optical power response, where higher hCG concentrations result in a lower normalized optical power response. This behavior is attributed to the LSP phenomenon.

**Figure 7:**
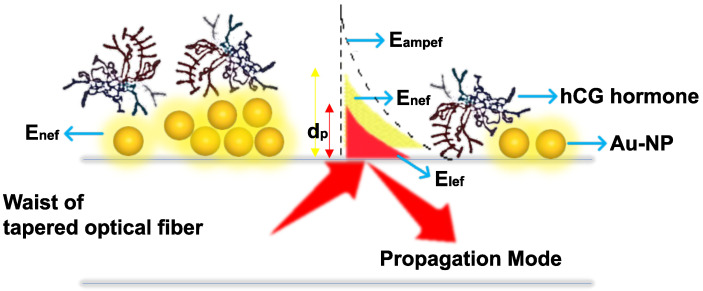
Illustrative scheme of the amplified evanescent field (E${}_{ampef}$) around the waist of the tapered optical fiber generated by the laser in the waist region (E${}_{lef}$), and the LSP of the Au-NPs (E${}_{nef}$).

The LSP creates a close electric field around the Au-NPs; when there is a constructive interaction between this field and the evanescent field in the waist of the tapered optical fiber, the evanescent electric field is amplified, as shown in Figure 7. This amplification enhances the sensitivity of the optical system, making it particularly effective for detecting low concentrations of the hCG hormone, as demonstrated in our experiments.

In this scheme, both the electric field generated by the laser and the evanescent field existing in the waist of the tapered optical fiber are presented in red (E${}_{lef}$). The near-electric field of the Au-NPs (E${}_{nef}$) is yellow. If there is a constructive interaction between both fields, resulting in an amplified field (E${}_{ampef}$) denoted by the curve with dotted lines, then the penetration depth (d${}_{p}$) of the resulting field in the surrounding also increases. This leads to a greater interaction with the molecules surrounding the tapered optical fiber, and as a result, a higher detection resolution of the hCG hormone is achieved.

It is considered that with higher concentrations of the hCG hormone, the interaction or energy absorption by the amplified evanescent fields increases, resulting in a decreased optical power response, and vice versa. The molecular groups responsible for absorbing the energy of the evanescent fields are those that compose the hCG hormone, particularly the C=O functional groups found in the lipids and proteins of the α and β units of the hCG hormone, as indicated in the characteristic FTIR spectrum.

It is important to mention that the amplification of the evanescent field leads to a greater interaction with the hCG hormone, even at low concentrations, as shown in the graph in Figure 6. However, according to our experimental results, it was observed that without the decoration of the tapered optical fiber with Au-NPs and with the same input power, no response is observed. To generate an evanescent field capable of interacting with the surrounding molecules and, thus, obtaining a power response, it would be necessary to apply a very high input power.

**Figure 8:**
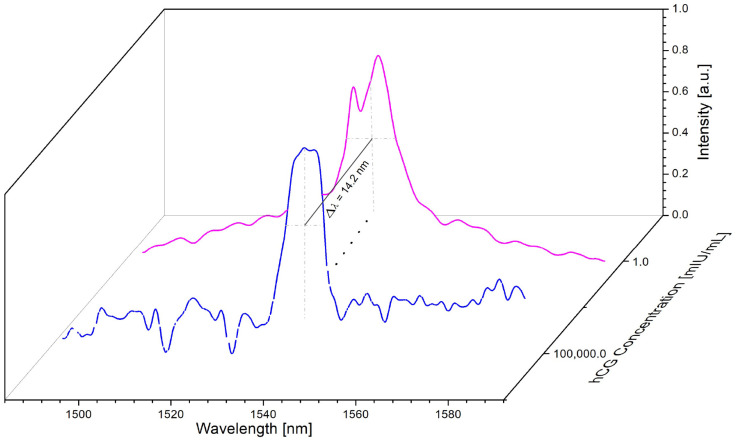
Wavelength shifts as a function of hCG concentrations.

The spectral analysis was achieved when depositing the different concentrations of the hCG hormone on the tapered optical fiber decorated with gold nanoparticles. Figure 8 presents the spectra for both the lower (1 mIU/mL) and higher (100,000 mIU/mL) concentrations of the hCG hormone.

In this case, a similar spectral behavior was observed as in previous reports [36,37]; there was a spectral shift as the hCG hormone concentration increased. A shift in wavelength from 1545.5 nm for a lower hCG concentration to 1531.3 nm for a higher hCG concentration was obtained. The spectral shift was 14.2 nm, and this is considered a result of the change in the refractive index of the surrounding medium. This spectral change supports the variation in power that occurs when the concentrations of the hCG hormone are varied in the solutions.

## 4. Conclusions

In this work, we carried out a study on the quantification of hCG hormone concentrations and their corresponding optical power responses using a tapered optical fiber decorated with Au-NPs. Our experimental results demonstrate an inverse relationship, where higher hCG hormone concentrations lead to diminished normalized optical power responses. This phenomenon arises from the LSP effect, which intensifies the electric field around 100 nm Au-NP-decorated tapered optical fibers with a diameter of 3 µm. Through the utilization of gold nanoparticles, this research successfully broadened the detection range and improved resolution, spanning a concentration range from 1 mIU/mL to 100,000 mIU/mL. Optical power responses exhibited distinct behaviors as hormone concentrations increased, driven by the absorption of evanescent fields by the hCG hormone. Notably, the study accurately quantified hCG concentrations as low as 1 mIU/mL, highlighting its potential for early pregnancy detection and the diagnosis of certain cancers, as these typically present with hCG hormone concentrations ranging from 16 mIU/mL to 40 mIU/mL.

## Data Availability

Not applicable.

## References

[1] Stenman U.H., Alfthan H., Hotakainen K. (2004). Human chorionic gonadotropin in cancer. Clin. Biochem..

[2] Dando I., Carmona-Carmona C.A., Zampieri N. (2021). Human chorionic gonadotropin-mediated induction of breast cancer cell proliferation and differentiation. Cells.

[3] Schüler-Toprak S., Treeck O., Ortmann O.O. (2017). Human chorionic gonadotropin and breast cancer. Int. J. Mol. Sci..

[4] Sheaff M.T., Martin J.E., Badenoch D.F., Baithun S.I. (1996). Beta hCG as a prognostic marker in adenocarcinoma of the prostate. Clin. Biochem..

[5] Venyo A.K., Herring D., Greenwood H., Maloney D.J. (2010). The expression of beta human chorionic gonadotrophin (*β*-HCG) in human urothelial carcinoma. Pan. Afr. Med. J..

[6] Hoshi S., Numahata K., Morozumi K., Katumata Y., Kuromoto A., Takai Y., Hoshi K., Bilim V., Sasagawa I. (2018). Bladder cancer metastasis producing beta-human chorionic gonadotropin, squamous cell carcinoma antigen, granulocyte-colony stimulating factor, and parathyroid hormone-related protein. IJU Case Rep..

[7] Syrigos K.N., Fyssas I., Konstandoulakis M.M., Harrington K.J., Papadopoulos S., Milingos N., Peveretos P., Golematis B.C. (1998). Beta human chorionic gonadotropin concentrations in serum of patients with pancreatic adenocarcinoma. Gut.

[8] Uesato Y., Kawamata F., Ishino S., Ono S., Tamashiro K., Koyama H., Takatsuki M. (2022). Human chorionic gonadotropin-*β* promotes pancreatic cancer progression via the epithelial mesenchymal transition signaling pathway. J. Gastrointest. Oncol..

[9] Liu F., Du F., Chen X. (2014). Multiple tumor marker protein chip detection system in diagnosis of pancreatic cancer. World J. Surg. Oncol..

[10] Manohar A., Werner E., Murphy J. (2012). Elevated Human Chorionic Gonadotrophin Due to Pancreatic Cancer. Open J. Anesthesiol..

[11] Zhao F., Zhu W., Su J., Xue Y., Wei W., Liu S. (2015). The enzyme linked immunosorbent and chemiluminescence assay for the detection of human chorionic gonadotrophin using soybean peroxidase as label enzyme. Curr. Anal. Chem..

[12] Chu C., Li L., Li S., Li M., Ge S., Yu J., Yan M., Song X. (2013). Fluorescence-based immunoassay for human chorionic gonadotropin based on polyfluorene-coated silica nanoparticles and polyaniline-coated Fe_3_O_4_ nanoparticles. Microchim. Acta.

[13] Lund H., Paus E., Berger P., Stenman U.H., Torcellini T., Halvorsen T.G., Reubsaet L. (2014). Epitope analysis and detection of human chorionic gonadotropin (hCG) variants by monoclonal antibodies and mass spectrometry. Tumour Biol..

[14] Suhito I.R., Koo K.M., Kim T.H. (2020). Recent Advances in Electrochemical Sensors for the Detection of Biomolecules and Whole Cells. Biomedicines.

[15] Zhang B., Mao Q., Zhang X., Jiang T., Chen M., Yu F., Fu W. (2015). A novel piezoelectric quartz micro-array immunosensor based on self-assembled monolayer for determination of human chorionic gonadotropin. Curr. Anal. Chem..

[16] Chen L., Leng Y., Liu B., Liu J., Wan S., Wu T., Yuan J., Shao L., Gu G., Fu Y. (2020). Ultrahigh-sensitivity label-free optical fiber biosensor based on a tapered singlemode- no core-singlemode coupler for Staphylococcus aureus detection. Sens. Actuators B Chem..

[17] Chen L., Liu B., Liu J., Wan S., Wu T., Yuan J., Zhou X., Long K., Shao L., Fu Y.Q. (2020). Novel Microfiber Sensor and Its Biosensing Application for Detection of hCG Based on a Singlemode-Tapered Hollow Core-Singlemode Fiber Structure. IEEE Sens. J..

[18] Belal S.J., Alameri L.M. (2019). Rashid, F.F.; Mansour, T.S. Laser biosensor as for pregnancy test by using photonic crystal fiber. Int. J. Med. Res. Health Sci..

[19] Chen C., Wang J. (2020). Optical biosensors: An exhaustive and comprehensive review. Analyst.

[20] Singh L., Agarwal N., Barthwal H., Arya B., Singh T., Huerta-Cuellar G. (2021). Application of Fiber Optics in Bio-Sensing. Fiber Optics.

[21] Lee B. (2003). Review of the present status of optical fiber sensors. Opt. Fiber Technol..

[22] Lyu S., Wu Z., Shi X., Wu Q. (2022). Optical Fiber Biosensors for Protein Detection: A Review. Photonics.

[23] Zeni L., Perri C., Cennamo N., Arcadio F., D’Agostino G., Salmona M., Beeg M., Gobbi M. (2020). A portable optical-fibre-based surface plasmon resonance biosensor for the detection of therapeutic antibodies in human serum. Sci. Rep..

[24] De Acha N., Socorro-Leránoz A.B., Elosúa C., Matías I.R. (2021). Trends in the Design of Intensity-Based Optical Fiber Biosensors 2010–2020. Biosensors.

[25] Tian Y., Wang W., Wu N., Zou X., Wang X. (2011). Tapered Optical Fiber Sensor for Label-Free Detection of Biomolecules. Sensors.

[26] Hamad E.M., Hawamdeh G., Jarrad N.A., Yasin O., Al-Gharabli S.I., Shadfan R. Detection of Human Chorionic Gonadotropin (hCG) Hormone using Digital Lateral Flow Immunoassay. Proceedings of the 40th Annual International Conference of the IEEE Engineering in Medicine and Biology Society (EMBC).

[27] Salim E.T., Fakhri M.A., Tariq S.M., Azzahrani A.S., Ibrahim R.K., Alwahib A.A., Alhasan S.F.H., Ramizy A., Salih E.Y., Salim Z.T. (2023). The Unclad Single-Mode Fiber-Optic Sensor Simulation for Localized Surface Plasmon Resonance Sensing Based on Silver Nanoparticles Embedded Coating. Plasmonics.

[28] Huo Z., Li Y., Chen B., Zhang W., Yang X., Yang X. (2023). Recent advances in surface plasmon resonance imaging and biological applications. Talanta.

[29] Nasirifar R., Danaie M., Dideban A. (2022). Highly sensitive surface plasmon resonance sensor using perforated optical fiber for biomedical applications. Optik.

[30] Esmailidastjerdipour P., Shahshahani F. (2023). Numerical Simulation of Surface Plasmon Resonance Optical Fiber Biosensor Enhanced by Using Alloys for Medical Application. Sens. Imaging.

[31] Gómez-Pavón L.C., Luis-Ramos A., González-Sierra N.E., Zaca-Morán P., Muñoz-Pacheco J.M., Coxca-Gutiérrez R.G. Design and fabrication of subwavelength optical fibers. Proceedings of the Latin America Optics and Photonics Conference.

[32] González-Sierra N.E., Gómez-Pavón L.C., Pérez-Sánchez G.F., Luis-Ramos A., Zaca-Morán P., Muñoz-Pacheco J.M., Chávez-Ramírez F. (2017). Tapered Optical Fiber Functionalized with Palladium Nanoparticles by Drop Casting and Laser Radiation for H2 and Volatile Organic Compounds Sensing Purposes. Sensors.

[33] Zandbaaf S., Khanmohammadi M., Garmarudi A., Rashidi B. (2020). Diagnosis of pregnancy based classification of embryo culture medium samples by infrared spectrometry and chemometrics. Infrared Phys. Technol..

[34] Xia N., Wang X., Liu L. (2016). A Graphene Oxide-Based Fluorescent Method for the Detection of Human Chorionic Gonadotropin. Sensors.

[35] Taha B.A., Ali N., Sapiee N.M., Fadhel M.M., Mat Yeh R.M., Bachok N.N., Al Mashhadany Y., Arsad N. (2021). Comprehensive Review Tapered Optical Fiber Configurations for Sensing Application: Trend and Challenges. Biosensors.

[36] Kumar R., Leng Y., Liu B., Zhou J., Shao L., Yuan J., Fan X., Wan S., Wu T., Liu J. (2019). Ultrasensitive biosensor based on magnetic microspheres enhanced microfiber interferometer. Biosens. Bioelectron..

[37] Meng X., Li S., Li J., Guo Y., Han Y., Wang Y., Gong L., Yuan J., Qiu S., Shen J. (2022). Refractive index sensing of twin-core fiber based on Mach Zehnder interference and SPR effect. J. Phys. D Appl. Phys..

